# Fusaric acid-evoked oxidative stress affects plant defence system by inducing biochemical changes at subcellular level

**DOI:** 10.1007/s00299-023-03084-9

**Published:** 2023-12-18

**Authors:** Nadeem Iqbal, Zalán Czékus, Attila Ördög, Péter Poór

**Affiliations:** 1 Department of Plant Biology, Faculty of Science and Informatics, University of Szeged, Közép Fasor 52, Szeged, 6726 Hungary; 2 Doctoral School of Environmental Sciences, University of Szeged, Szeged, Hungary; 3 Doctoral School of Biology, University of Szeged, Szeged, Hungary

**Keywords:** Fusaric acid, Oxidative stress, Cell death, Antioxidants, Phytohormones

## Abstract

Fusaric acid (FA) is one of the most harmful phytotoxins produced in various plant–pathogen interactions. *Fusarium* species produce FA as a secondary metabolite, which can infect many agronomic crops at all stages of development from seed to fruit, and FA production can further compromise plant survival because of its phytotoxic effects. FA exposure in plant species adversely affects plant growth, development and crop yield. FA exposure in plants leads to the generation of reactive oxygen species (ROS), which cause cellular damage and ultimately cell death. Therefore, FA-induced ROS accumulation in plants has been a topic of interest for many researchers to understand the plant–pathogen interactions and plant defence responses. In this study, we reviewed the FA-mediated oxidative stress and ROS-induced defence responses of antioxidants, as well as hormonal signalling in plants. The effects of FA phytotoxicity on lipid peroxidation, physiological changes and ultrastructural changes at cellular and subcellular levels were reported. Additionally, DNA damage, cell death and adverse effects on photosynthesis have been explained. Some possible approaches to overcome the harmful effects of FA in plants were also discussed. It is concluded that FA-induced ROS affect the enzymatic and non-enzymatic antioxidant system regulated by phytohormones. The effects of FA are also associated with other photosynthetic, ultrastructural and genotoxic modifications in plants.

**Graphical abstract:**

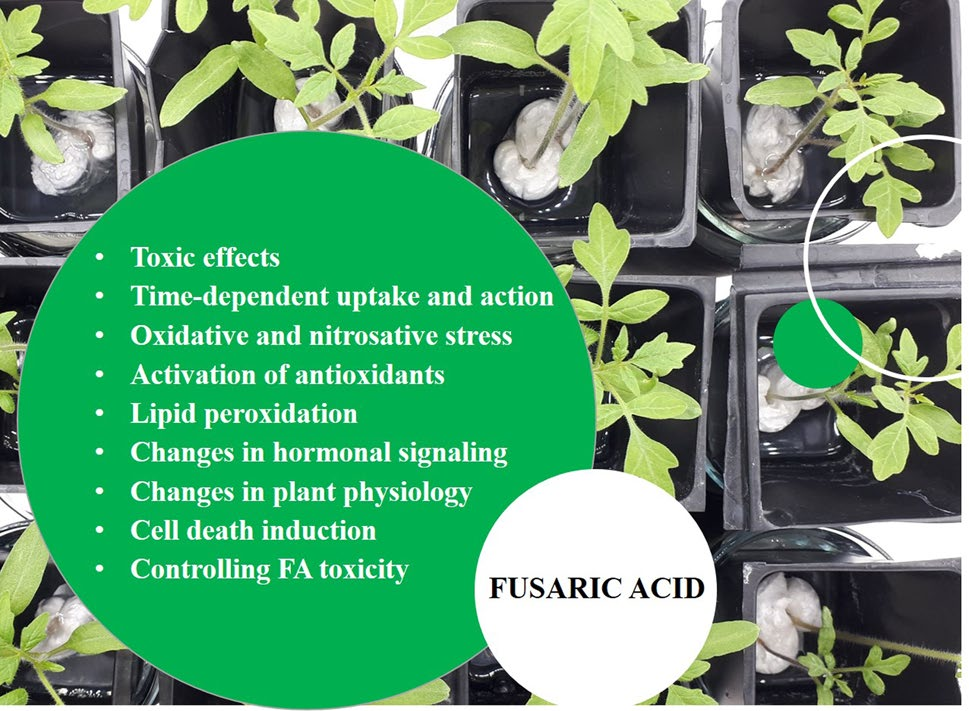

## Introduction

### *Fusarium* toxins and their exposure

Phytotoxins are produced by several fungal species as secondary metabolites that can have severe toxic effects on plants and animals, including humans (Zain 2011). Every year, significant crop yields are lost because of the detrimental effects of phytotoxins, e.g. *Fusarium* toxins, on agricultural crops worldwide (Ismaiel and Papenbrock 2015; Pleadin et al. 2019; Rodríguez-Carrasco et al. 2013). These *Fusarium* toxins are low molecular weight compounds that are produced under many environmental conditions, mainly in cereals at all stages of development (Rodríguez-Carrasco et al. 2013). Therefore, these *Fusarium* toxins can contaminate many food sources and cause serious health problems when consumed (Alshannaq and Yu 2017; Iqbal et al. 2021a). The accumulation of these phytotoxins in the food chain is a major problem that is receiving considerable attention at the global level (Zain 2011; Rodríguez-Carrasco et al. 2013). Phytotoxin contamination in food and feed occurs as a result of malnutrition, poor handling techniques, inappropriate food storage and consumption of staple foods obtained from toxin-contaminated sites (Milićević et al. 2010). *Fusarium* toxins have been reported as carcinogens, hepatotoxins, nephrotoxins and neurotoxins (Arumugam et al. 2021). At the same time, the nature and damage caused by these phytotoxins vary with the type of phytotoxin, concentration, exposure time, route of consumption, health status and sex of the affected person (Zain 2011). Humans are mainly exposed to these toxins through the consumption of contaminated food and transfer of phytotoxins in animal products. In addition to oral ingestion of fungal contaminated food (Pinotti et al. 2016), skin contact (dermatophyte skin infection) and inhalation of spores (toxic pneumonitis) are also routes of exposure to *Fusarium* toxins (Kuruvilla and Dias 2012). Fusaric acid (FA), fumonisins, trichothecenes, zearalenone, deoxynivalenol, moniliformin, fusaproliferin, enniatins and beauvericin are classified as the most common and harmful phytotoxins (Arumugam et al. 2021; Ismaiel and Papenbrock 2015).

### Basic overview of fusaric acid

**Fig. 1:**
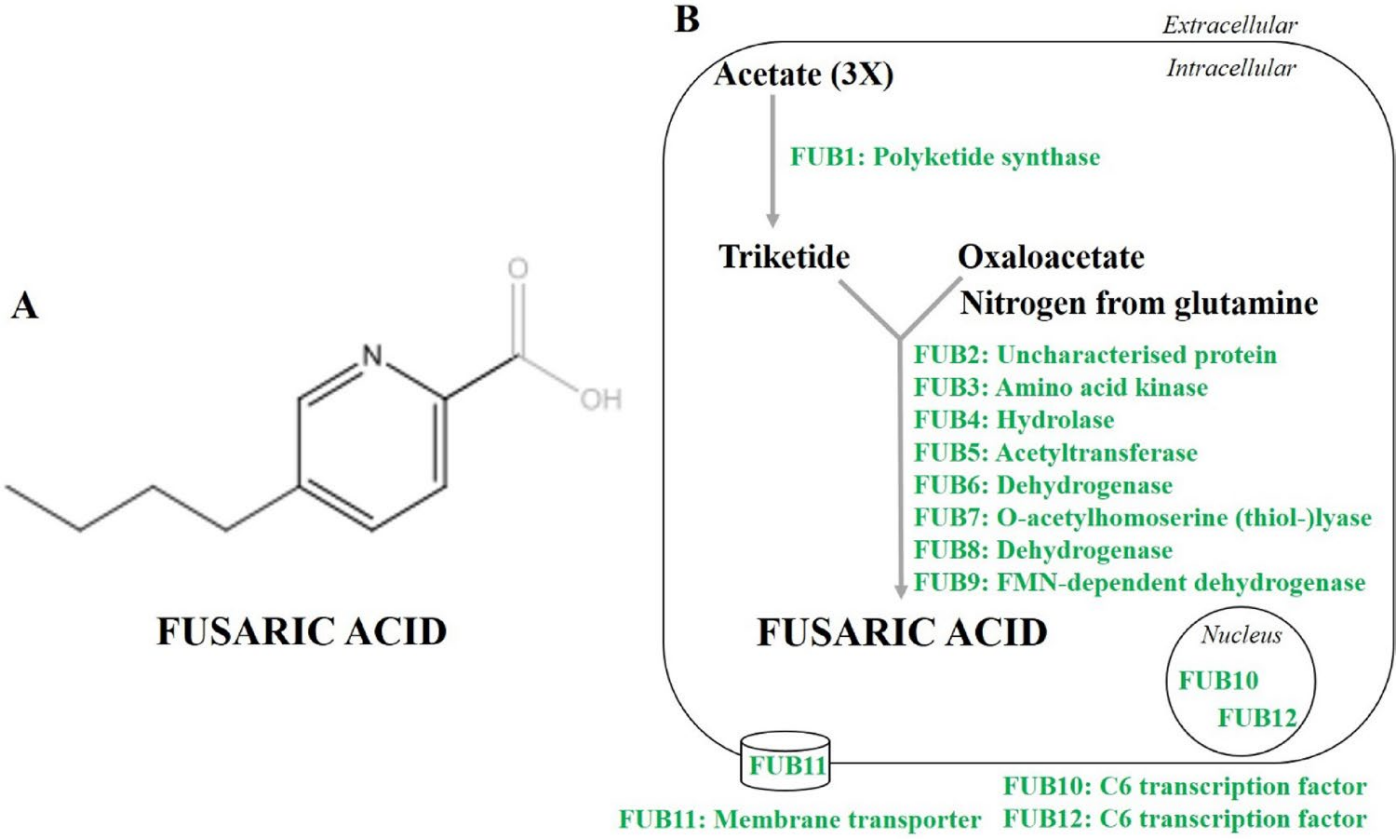
Chemical structure of fusaric acid (FA) (**A**) and the biosynthetic pathway of FA in *Fusarium* species (**B**) (based on Brown et al. 2012, 2015; Niehaus et al. 2014; Sánchez-Rangel et al. 2018)

FA is one of the most harmful and non-specific phytotoxins produced by various *Fusarium* species. The molecular formula and weight of FA are C_10_H_13_NO_2_ and 179.2157 g/mol, respectively (Singh et al. 2017). FA has been recognized as a phytotoxin involved in the development of plant diseases. During the separation of gibberellins in 1934, a Japanese scientist found a crystalline compound from the fungus *Gibberella fujikuroi*, which was later named FA (Brown et al. 2012; Sieber et al. 2013). FA is a derivative of picolinic acid (5-butylpicolinic acid; Fig. 1A) which is known as a metal chelating agent (Ismaiel and Papenbrock 2015). FA production is not restricted to *Gibberella fujikuroi*, but the entire *Fusarium* genus has the ability to produce it (Brown et al. 2012; Sieber et al. 2013). Interestingly, the 5-butyl side chain of FA is involved in enhancing the lipophilicity of FA which assists in the penetration through the cell membrane (Devnarain et al. 2017). In addition, FA has a carboxylic acid group, which acts as a proton donor and is responsible for its acidic properties in various reactions (Liu et al. 2016). As a result of the chelate-forming ability of FA, it binds with other divalent cations, causing the conjugation of iron, manganese, copper and zinc, which hinders their ability to perform biological functions (e.g. as cofactors of the antioxidant enzyme superoxide dismutase) (Arumugam et al. 2021).

### FA-producing fungal species

Besides the FA-producing *Fusarium fujikuroi*, previously known as *Gibberella fujikuroi*, there are 50 to 100 phylogenetically closely related *Fusarium* species with the ability to produce FA such as *F*. *verticillioides* and *F. proliferatum* (Cen et al. 2020). Among the other closely related *Fusarium* species that are able to produce FA we can find *F. oxysporum*, *F. mangiferae*, *F. subglutinans*, *F. circinatum* and the remotely related *F. solani*, *F. napiforme* and *F. crookwellense* (Ismaiel and Papenbrock 2015). The high production of FA depends on the virulence of the fungal strain and the susceptibility of the host plants, such as banana, cucumber, potato, tobacco, tomato and wheat (Alshannaq and Yu 2017; Singh et al. 2017). Briefly, a virulent fungal strain can produce a higher amount of FA, resulting in more severe plant infection, whereas a susceptible plant with a weak immune system cannot resist fungal attack, resulting in more severe fungal infection (Singh et al. 2017).

### FA biosynthesis

FA is a polyketide-derived secondary metabolite (Srivastava et al. 2020). The genes responsible for its biosynthesis are located next to each other in the genome. The genomic cluster for FA production involves genes encoding specific enzymes, transport proteins and transcription factors (Brown et al. 2015). Previous studies have reported that the FA biosynthesis gene cluster (*FUB*) comprises five genes in *F. verticillioides* (maize pathogen) and *F. fujikuroi* (rice pathogen) (Brown et al. 2012; Niehaus et al. 2014). However, transcription factors and transporter genes were not included in the cluster (Brown et al. 2015). Subsequently, the FUB region was analysed in several *Fusarium* species such as *F. verticillioides*, *F. fujikuroi* and *F. oxysporum.* A total of 12 genes (*FUB1* to *FUB12*) are present in the FUB cluster (Brown et al. 2015). Furthermore, deletion analysis revealed that nine *FUB* genes and two transcription factors (Zn(II)_2_Cys_6_) are responsible for FA production (Brown et al. 2015). In one research study, the homologs of the FUB gene cluster were compared with the gene clusters of other *Fusarium* species, and it was found that certain homologs had non-FUB genes inserted at specific positions (Brown et al. 2012, 2015). The *F. verticillioides* and *F. oxysporum* infection of maize and cactus plants showed that FA production contributes to the phytotoxicity of *Fusarium* species but did not affect the virulence of the fungus (Brown et al. 2015). A proposed pathway for FA production in *Fusarium* sp. is shown in Fig. 1B. In this pathway, polyketide synthase (FUB1) converts three molecules of acetate to triketide, which further reacts with oxaloacetate to form FA. Nitrogen supply required for FA synthesis is derived from glutamine (Brown et al. 2015). The predicted functions of the FUB clusters were revealed by Brown and co-workers (2015) in *F. verticillioides* and *F. oxysporum*. As a result of the lack of distinct FA intermediates in fungal *fub* mutant cultures, the exact functions of the *FUB3*, *FUB4*, *FUB5*, *FUB6* and *FUB8* genes could not be assigned (Brown et al. 2015; Niehaus et al. 2014). Therefore, the exact role of the polyketide precursor in FA synthesis is still unknown. Similarly, *FUB2* is assigned to encode a small protein, but its function is also currently unknown (Brown et al. 2015). However, *FUB7* and *FUB9* encode a sulfhydrylase and an oxidase, respectively, but their specific role in FA biosynthesis is unclear (Brown et al. 2015). Both *FUB10* and *FUB12* genes encode C6 transcription factors, while *FUB11* acts as a transporter-encoding gene (Brown et al. 2015). FUB6, FUB8 and FUB10, which are responsible for FA production, may provide the basis for future research regarding the regulation of FA synthesis in *Fusarium* species. FB10 is a positive regulator of the other gene clusters while FB11 acts as a major facilitator superfamily (MFS) which plays a role in detoxification processes by removing excess FA (Studt et al. 2016). Moreover, FUB12 is also involved in the detoxification of FA to dehydrofusaric acid and then to fusarinolic acid by regulating the expression of cytochrome P450 (Studt et al. 2016). On the basis of the available literature, controlling the expression of *FB6*, *FB8* and *FB10* genes may lead to the development of novel strategies to limit FA production (Brown et al. 2015).

## Toxicity of FA and its mechanism

### Toxicity of FA in animals, humans and other organisms

Consumption of contaminated plant-derived foods can result in the accumulation of mycotoxins in body cells of both humans and animals, where FA concentrations can reach dangerous levels up to 10^−5^ M (da Rocha et al. 2014; Sobral et al. 2018). Higher levels of FA are carcinogenic to the human immune system, reproductive organs, liver, kidney and brain (da Rocha et al. 2014; Mamur et al. 2020). For instance, FA toxicity in human hepatocellular carcinoma (HepG2) cell line induced DNA damage and post-translational modifications of p53 (a tumour suppressor protein) by increasing the activity histone deacetylases and inhibiting histone acetyltransferases followed by increased cell proliferation and apoptosis in the cells (Ghazi et al. 2017). In addition, FA induces skin and gastrointestinal disorders and alters gene function leading to abnormal embryo development (Mézes 2008). FA toxicity can inhibit the activity of dopamine β-hydroxylase, which is responsible for the proper functioning of the nervous system (Reddy et al. 1996). FA has been shown to be cytotoxic to human normal fibroblast as well as colon and breast cancer cells at concentrations of 500 µM (Fernandez-Pol et al. 1993). In another study, Mamur and co-workers (2020) analysed the toxic effects of FA on human lymphocytes and human cervical carcinoma cells, and showed that FA at a concentration of 400 µg/mL had both cytotoxic and genotoxic effects. FA also induced toxic effects on rat hepatoma cells, Chinese hamster ovary, mouse fibroblast and dog kidney fibroblast cells (Mamur et al. 2020; Vesonder et al. 1993). FA also induced toxic effects on zebrafish embryo development by chelating the active site of lysyl oxidase (an enzyme essential for respiratory function, ovulation and wound healing) with copper to inhibit its activity, resulting in teratogenic effects (Yin et al. 2015).

In addition to animals and humans, FA also affected bacterial growth (10^−4^ to 10^−3^ M), cell wall permeability in green algae (*Spirogyra nitida*) (5 × 10^−3^ M) and spore germination of the fungus *Ustilago maydis* at 1.5 × 10^−4^ M concentration (Srivastava et al. 2020). Therefore, more preventive measures are needed to reduce the toxic effects of FA in feed and food.

### FA toxicity in plants

**Fig. 2:**
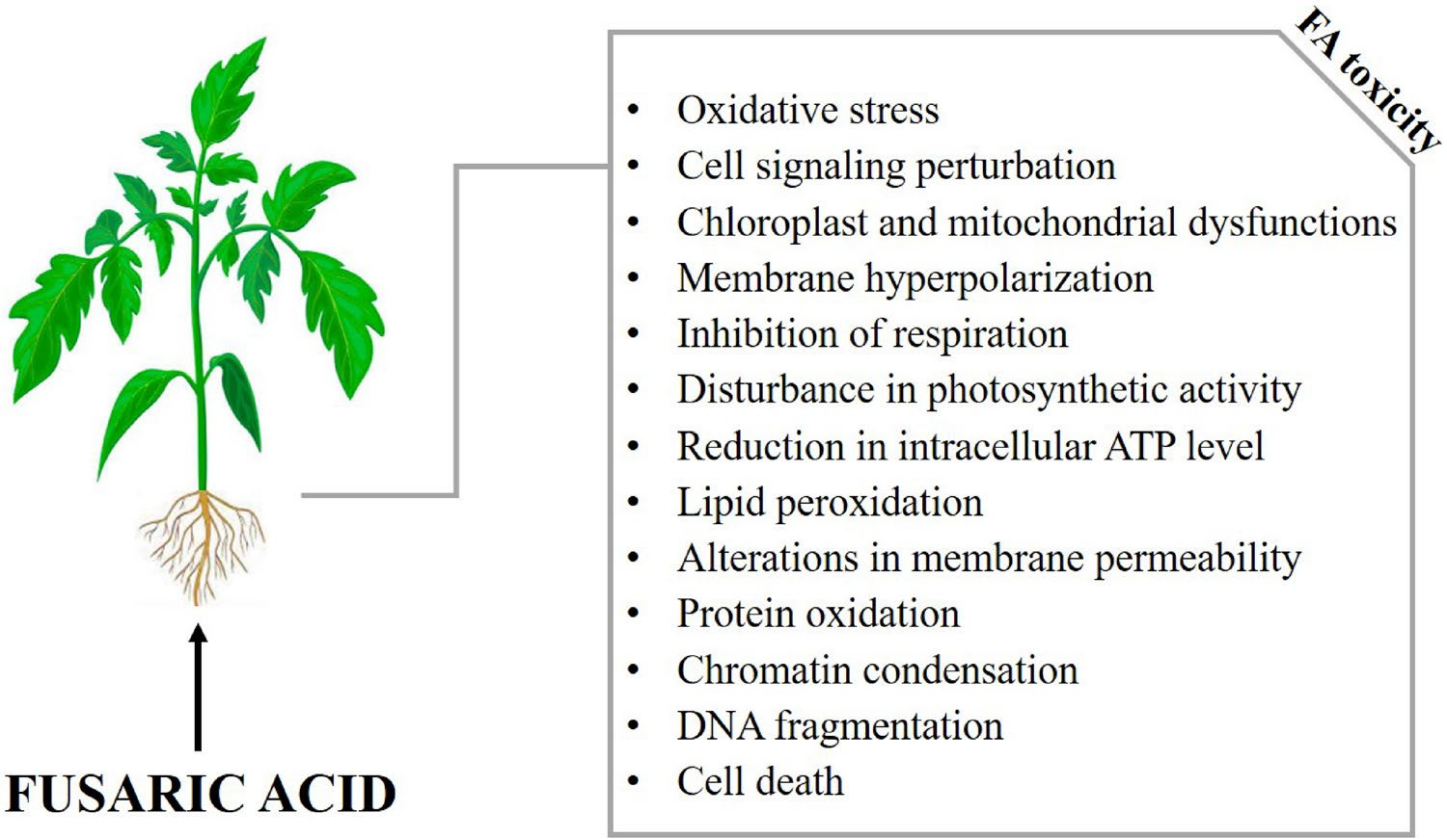
General toxicity symptoms of fusaric acid (FA) in plants

FA also causes plant diseases such as fusarium wilt, which reduces the yield of many agricultural crops (Singh et al. 2017). The toxicity of FA has been documented in many plant species such as tomato (Singh et al. 2017), potato (Sapko et al. 2011), cucumber (Wang et al. 2015), watermelon (Wu et al. 2007), banana (Fung et al. 2019), date palm (Bouizgarne et al. 2004), *Arabidopsis* (Bouizgarne et al. 2006a), maize (Spss and Oliveira 2009), cotton (Stipanovic et al. 2011), tobacco (Jiao et al. 2014), wheat (Li et al. 2021), barley (Liu et al. 2016), saffron (Samadi and Shahsavan Behboodi 2006), wax gourd (Wang et al. 2021), chihuahua flower (Antić et al. 2020), faba bean (Li et al. 2021), castor bean (Pavlovkin et al. 2004), cape gooseberry (Mendoza-Vargas et al. 2021), sword lily (Nosir et al. 2011) and hemp broomrape (Bouizgarne et al. 2006b). In plants, FA induces oxidative stress, chloroplast and mitochondrial dysfunction, lipid peroxidation, protein damage and DNA fragmentation (Jiao et al. 2014; Iqbal et al. 2021a) (Fig. 2). FA causes wilting, necrotic spots on leaves, reduction in roots and root hair growth (Singh et al. 2017), cell signalling perturbation, disruption of photosynthetic activity (Iqbal et al. 2021a), inhibition of respiration, membrane hyperpolarization and reduction in intracellular ATP levels (Bouizgarne et al. 2006a). FA has also been reported to inhibit cytochrome oxidase activity, alter membrane permeability, induce plasma membrane damage, chromatin condensation and electrolyte leakage (Kuźniak 2001; Singh and Upadhyay 2014).

In addition, FA can exert its phytotoxic effect on plant species in the dose range of 20–200 mg/kg (Manda and Srivastava 2021). Previous research results suggest that the FA concentration is also improved by the conversion of dehydrofusaric acid to FA (Manda and Srivastava 2021). Exposure of plants to FA results in chlorosis, chelation of copper and iron, changes in the cell wall permeability, disturbance of ionic balance as well as inhibition of enzymatic processes such as via the defectiveness of respiratory enzymes (Srivastava et al. 2020). In addition to FA, other phytotoxins have also caused similar wilt symptoms in plants, which require further investigation.

### FA uptake and mechanism of action in plants

Many studies have reported the phytotoxicity and disease symptoms caused by FA in plants; however, some recent studies also investigated the mechanism of action of FA in plants (López–Díaz et al. 2018; Iqbal et al. 2023). FA induces wilt symptoms in tomato seedlings, when applied via the rooting medium (root uptake). The wilting characteristics in tomato cotyledons and lower leaves indicate the ability of FA to spread throughout the plant (López–Díaz et al. 2018). In the cucumber and banana plants, transport of FA from the roots to the upper parts (shoot, stem, leaf) was observed via both xylem and phloem pathways (Wang et al. 2013; Dong et al. 2014). The transport of FA within the plants causes membrane damage to the transport vessels which eventually leads to wilting (Dong et al. 2014; Wang et al. 2013). These findings are also consistent with another study where wilting symptoms were observed in watermelon seedlings (Wu et al. 2008a). However, Ruiz and co-workers (2015) suggested that the movement of FA within the plants was due to its chelating ability for metal ions which induces siderophore synthesis. Furthermore, the application of metal ions such as copper, zinc or iron improved the chelating ability of FA but at the same time inhibited the phytotoxicity of FA in tomato plants (López–Díaz et al. 2018). The inhibition of FA toxicity was also functional when FA and metal ions were applied to different plant parts such as leaves and roots, demonstrating that the chelating mechanism occurs in plants (López–Díaz et al. 2018). Furthermore, the presence of membrane-permeable TPEN (metal chelator) showed toxicity similar to that of FA (Fernandez-Pol et al. 1993). Although other possible mechanisms cannot be excluded, these results accurately elucidate the relationship between metal chelation and FA toxicity. *Fusarium* infection-induced disease symptoms in banana plants were further exacerbated by FA production, leading to acceleration of senescence (Dong et al. 2014). During this process, FA directly damaged the cell membranes and chloroplasts and then accumulated in the lower leaves to enhance senescence, demonstrating that FA can have both direct and indirect toxic effects in plants (Dong et al. 2014).

## FA-induced oxidative and nitrosative stress in plants

FA can induce ROS production at or above 10^−5^ M concentration in different plant species (Singh and Upadhyay 2014). ROS include hydrogen peroxide (H_2_O_2_), superoxide radical (O_2_^**.**−^), hydroxyl ion (^−^OH) and singlet oxygen (^1^O_2_) (Singh and Upadhyay 2014). FA-mediated ROS accumulation is a major contributor to the development of disease symptoms in plants (Singh et al. 2017). It is well known that pathogenic attacks increase ROS production, which ultimately triggers the plant hypersensitive response (HR). HR is part of plant cell death that limits the spread of fungal infection by affecting many organelles such as mitochondria, chloroplast and nucleus (Singh et al. 2017; Singh and Upadhyay 2016; Karuppanapandian et al. 2011). Among ROS, H_2_O_2_ is a long-lasting molecule that plays an important role in plant immunity against pathogens, and acts as a signalling molecule under phytotoxin exposure (Zaynab et al. 2019). The control and regulation of ROS production as well as degradation was observed to be carried out by various antioxidant mechanisms (Ali et al. 2018). In addition, beneficial effects of ROS on cell functions such as growth and differentiation have also been reported at lower concentrations, but higher concentrations may be detrimental to cells by inducing cell death (Ma 2013). Under FA exposure, ROS accumulation affects many biochemical processes (Breusegem and Dat 2006). The balance between ROS production and ROS scavenging can be a determining factor for the infection of plant pathogens. Exposure to phytotoxins can perturb this redox balance and lead to ROS accumulation resulting in toxicity and disease (Arumugam et al. 2021; Mates 2000). At the same time, FA-induced ROS generation in different plant species has not been well studied and further research is needed to understand the exact mechanism of ROS production in the plant cell.

Under phytotoxin exposure, ROS interact with phytohormones to induce cell death (Overmyer et al. 2005). In addition, ROS production can cause numerous changes in the cellular components such as inactivation of enzymes, degradation of proteins and nucleic acids, and lipid peroxidation (Maksymiec and Krupa 2006). The contribution of ROS overproduction in plants to FA phytotoxicity has been a topic of interest for researchers (Malerba and Cerana 2021; Liu et al. 2020). Different concentrations of FA have been used in plants to induce ROS accumulation and to study their toxic effects on cellular functions (Sapko et al. 2011; Bouizgarne et al. 2006a). The results showed a time- and concentration-dependent phytotoxic effect of FA. For example, treatment of tomato plants with FA (0.1–1 mM) significantly increased ROS production after 24–72 h, and this oxidative burst damaged cellular functions and photosynthetic activity as well (Iqbal et al. 2021a). Similarly, FA doses of 50–300 µg/mL induced ROS generation (O_2_^**.**−^ and H_2_O_2_) in tomato leaves after 72 h of treatment (Singh et al. 2017; Singh and Upadhyay 2014, 2016). Similarly, FA concentrations in the range of 10^−8^–10^−3^ M induced H_2_O_2_ production in cell suspension of potato plants after 48 h of exposure (Sapko et al. 2011). In comparison, various FA concentrations caused ROS accumulation (H_2_O_2_) and disrupted the cellular processes in many agronomic crops such as tobacco (Jiao et al. 2014), cucumber (Wang et al. 2020) and *Arabidopsis* (Bouizgarne et al. 2006a). In addition, high ROS (O_2_^**.**−^, H_2_O_2_) accumulation was also observed in saffron (Samadi and Shahsavan Behboodi 2006), tomato (Kuźniak 2001) and banana plants (Fung et al. 2019) under different FA doses, where the duration of FA exposure determined its effect on plant growth and development. Fung et al. (2019) reported rapid accumulation of both O_2_^**.**−^ and H_2_O_2_ during FA-producing fungal infection at the early stage in banana plants. At the same time, higher ROS generation did not slow down the fungal infection, indicating that the accumulated ROS were not sufficient to prevent fungal pathogenicity (Fung et al. 2019). However, fungal tolerance to higher ROS production is still unknown. Interestingly, another study reported higher production of O_2_^**.**−^ in culture medium (extracellular or filtrate) as compared to cellular extracts of tomato separated from culture medium (Kuźniak 2001). Nevertheless, H_2_O_2_ production showed the same levels in the culture medium and cellular extract upon FA treatment as compared to their respective controls (Kuźniak 2001). In addition, increased O_2_^**.**−^ production was detected in the culture medium 12 h after the FA exposure and increased H_2_O_2_ production was measured 48 h after the treatment in cellular extracts (Kuźniak 2001). Therefore, these results clearly indicate that rapid or gradual induction of ROS production is directly proportional to the dose and exposure time of FA. The results also suggest that ROS accumulation may vary between plants and even within cells or extracellular spaces.

FA stress induced plasmolysis, cytoplasmic vacuole formation and cytoplasmic shrinkage in tobacco cell suspensions (Jiao et al. 2013). Interestingly, nitric oxide (NO) scavengers significantly reduced these FA-induced toxic effects in tobacco cells (Jiao et al. 2013). The *PAL* (phenylalanine ammonia-lyase) and *Hsr203J* (hypersensitivity-related protein) genes were investigated for their potential role in regulating plant–pathogen interactions and enhancing plant immunity against fungi or their phytotoxins (Jiao et al. 2013). In addition, the NO scavenger 2-(4-carboxyphenyl)-4,4,5,5-tetramethylimidazoline-1-oxyl-3-oxide (CPTIO) also reduced the caspase-3-like activity and the expression of *PAL* and *Hsr203J* genes, indicating the crucial role of the NO signalling pathway in the regulation of plant cell death under FA exposure (Jiao et al. 2013). Therefore, the application of NO scavenger can also significantly reduce FA-induced phytotoxic effects and promote plant growth and development, confirming the significant role of NO under FA exposure.

## FA-induced changes in enzymatic and non-enzymatic antioxidants

**Fig. 3:**
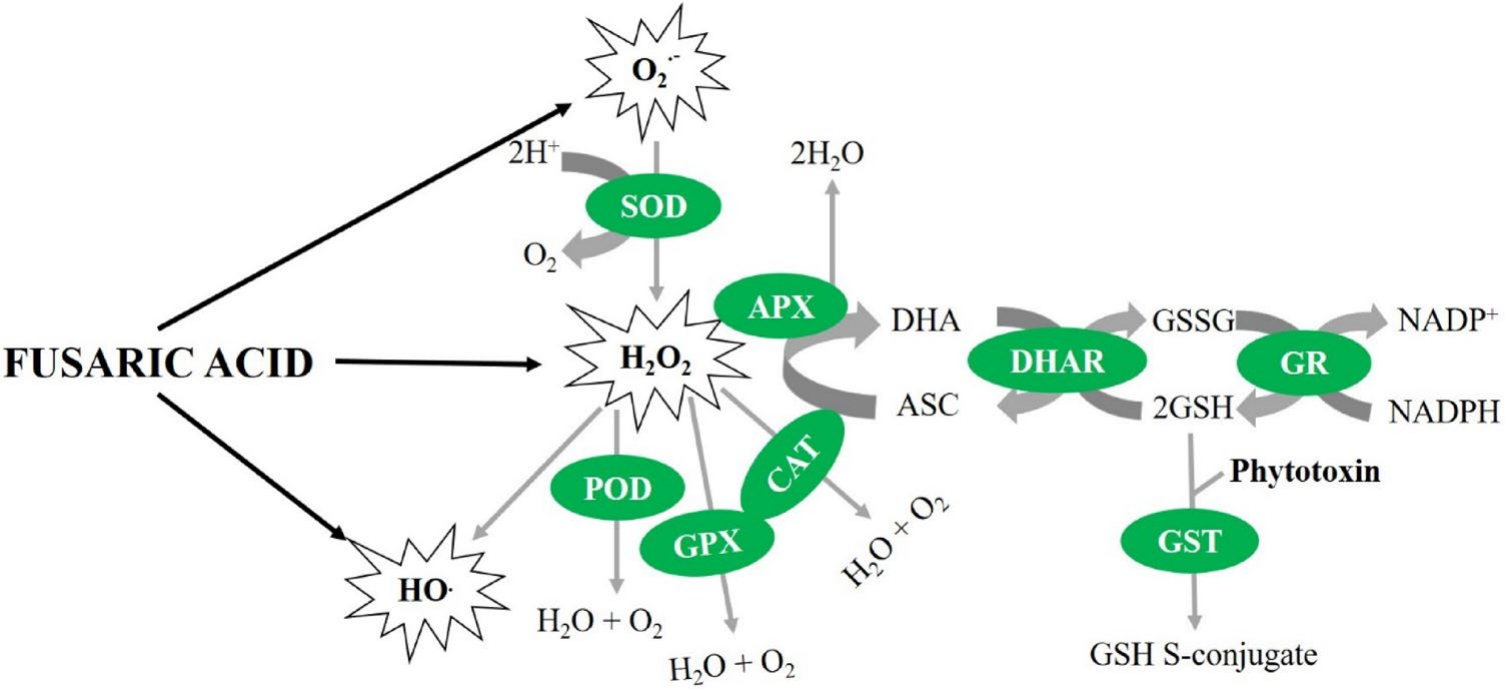
Mechanism of ROS detoxification by enzymatic and non-enzymatic antioxidants under fusaric acid (FA) exposure. *CAT* catalase, *SOD* superoxide dismutase, *POD* peroxidase, *GST* glutathione S-transferase, *GPX* glutathione peroxidase, *APX* ascorbate peroxidase, *GR* glutathione reductase, *DHAR* dehydroascorbate reductase, *GSH* glutathione, *ASC* ascorbate

Plants contain both enzymatic and non-enzymatic antioxidants to attenuate ROS accumulation under phytotoxin FA exposure (Fung et al. 2019; Singh et al. 2017). These ROS scavengers maintain ROS homeostasis in the cells by removing ROS and play an important role in oxidative metabolism. ROS accumulation upon FA exposure induces both enzymatic and non-enzymatic antioxidants (Antić et al. 2020; Dong et al. 2014). These ROS-scavenging antioxidants include superoxide dismutase (SOD), catalase (CAT), ascorbate peroxidase (APX), peroxidase (POD), glutathione peroxidase (GPX), glutathione reductase (GR), glutathione S-transferase (GST), dehydroascorbate reductase (DHAR), glutathione (GSH), ascorbate (ASC) and phenolic compounds (Sharma et al. 2022; Hashem et al. 2021; Gupta et al. 2018; Sapko et al. 2011; Foyer and Noctor 2009). These antioxidants are responsible for cell protection and defence against toxic levels of overproduced ROS following fungal attacks (Gill and Tuteja 2010). SOD catalyses the dismutation of O_2_^**.**−^ radicals to O_2_ and H_2_O_2_. Similarly, CAT and APX catalyse the conversion of H_2_O_2_ molecules into water and oxygen molecules. POD is responsible for the conversion of H_2_O_2_ and dangerous hydroperoxides (ROOH) to water and harmless ROH species. Similarly, GR can increase reduced GSH levels under oxidative stress and GST can enhance plant resistance to fungal pathogens by conjugating GSH with their toxins for the process of detoxification (Fung et al. 2019; Gullner et al. 2018; Liu et al. 2015). GPX can convert H_2_O_2_ to water and oxygen, and peroxide radicals to alcohol and oxygen molecules, while DHAR is involved in the conversion of dehydroascorbic acid to ascorbic acid (Loi et al. 2020). Nevertheless, further scientific research is needed to understand the role of these ROS-scavenging compounds under phytotoxin exposure, and their involvement in alleviating phytotoxin-induced oxidative damage in plants. The mechanisms of different ROS-scavenging antioxidants under FA exposure are summarized in Fig. 3.

FA-induced oxidative stress affected antioxidant levels in several plant species. For example, treatment with 100 µM FA reduced APX and CAT activities in tobacco cell suspension culture after 24 h (Jiao et al. 2014). Similarly, Hashem et al. (2021) infected tomato plants with the FA-producing fungus *F. oxysporum* and then observed that the activities of the enzymes DHAR and GPX decreased, while the enzymatic activities of SOD and APX increased as a result of the fungal infection. Conversely, higher SOD (after 48 h) and CAT (after 12 and 24 h) activities were significantly increased after FA treatment (2 × 10^−4^ M) in cell suspensions of tomato plants (Kuźniak 2001). The same trend was observed for other antioxidants such as APX and POD. However, DHAR activity and ASC content were reduced under FA stress in a time-dependent manner (Kuźniak 2001). Similarly, different concentrations of FA treatments (0–400 mg/L) resulted in initially enhanced activities of SOD, POD and CAT in watermelon leaves, which then gradually decreased in a time-dependent manner in a 24-h-long experiment (Wu et al. 2008b). Similarly, banana seedlings infected with an FA-producing fungus showed significantly higher activities of GST, SOD, POD, CAT, GR and APX initially, which then gradually decreased in a 10-day-long experiment (Fung et al. 2019). Different concentrations of FA or FA-producing fungal species were used to determine the detoxification capacities of different enzymatic and non-enzymatic antioxidants in several plant species such as cucumber (Wang et al. 2020), potato (Sapko et al. 2011), wax gourd (Wang et al. 2021) and chihuahua flower (Antić et al. 2020). Interestingly, tomato leaves showed lower activities of CAT and APX due to the possible inhibitory effect of FA on these ROS scavengers, but SOD activity was increased with increasing time duration to detoxify ROS overproduction (Singh and Upadhyay 2014). Another study showed higher peroxidase (POX) activity due to its involvement in the cross-linking of cell wall proteins (Singh and Upadhyay 2016). FA treatment elevated the level of H_2_O_2_ which was detoxified by the POX enzyme. Furthermore, FA exposure decreased POD and CAT activities in wheat and faba bean (intercropping) plants, as the FA concentration increased from 0 to 200 mg/L (Li et al. 2021); however, the highest POD activity and the highest level of total phenolic compounds were found in banana plants during fungal infection due to FA production (Dong et al. 2014). These FA-induced changes in the antioxidant system could affect how plants respond by increasing or decreasing the amount of antioxidants produced, which can result in either plant survival or cell death. In addition, FA can have an inhibitory effect on antioxidant activities and higher levels can kill plant cells (Wu et al. 2008b; Singh and Upadhyay 2014). Therefore, a potent antioxidant system plays a critical role in detoxifying ROS produced in cellular compartments upon exposure to fungi or phytotoxin FA. The antioxidant system promotes plant growth by enhancing plant defence responses against phytotoxins such as FA. It is also noteworthy, that FA-induced changes in antioxidant activities are dependent on plant species, FA concentration, the examined plant organ and FA exposure time.

## FA-mediated lipid peroxidation

As a fundamental component of biological membranes, lipids play a vital role in cellular function. FA-induced oxidative stress can damage lipids and disrupt the proper functioning of cells. ROS attack the carbon–carbon double bonds in polyunsaturated fatty acids, generating hydroperoxides and peroxyl radicals (Ayala et al. 2014). The disturbance of the plasma membrane caused by the lipid peroxyl radicals’ accelerated peroxidation alters the fluidity and permeability of the membrane, disrupts ion transport and interferes with cell signalling (Birben et al. 2012). In addition, 4-hydroxynonenal (HNE) and malondialdehyde (MDA) are reactive products of lipid peroxidation and are considered as indicators of oxidative stress (Arumugam et al. 2021).

Lipid peroxidation by FA exposure depends on the exposure time and the concentration of the phytotoxin. FA concentration between 50 and 200 mg/L resulted in lipid oxidation, and significantly increased the MDA content in wheat and faba bean seedlings (Li et al. 2021). Similarly, FA exposure at a concentration of 100 mg/L increased the MDA level in cape gooseberry plant after 12 h (Mendoza-Vargas et al. 2021). Further, other studies also showed that exposure of watermelon to FA (0–400 mg/L) increased MDA levels at 24 h. The MDA content significantly increased with the duration of FA exposure, reaching a maximum (5–11-fold) at 24 h post treatment as compared to control plants (Wu et al. 2008b). Interestingly, MDA is expressed as thiobarbituric acid-reacting substances (TBARS) (Singh et al. 2017). Therefore, numerous studies have been conducted on FA-induced oxidative stress leading to lipid peroxidation, and increased MDA and TBARS levels in the FA-treated plants. For example, FA (200 mg/L) significantly reduced the MDA levels in the roots of wax gourd during a 48-h-long experiment (Wang et al. 2021). In contrast, lipid peroxidation caused by FA treatment (100 µM) resulted in higher MDA levels (threefold) in tobacco cell suspension at 12 and 24 h; however, no rapid MDA generation was observed after treatment for 3 and 6 h (Jiao et al. 2014). In parallel, MDA levels in cucumber, banana, tomato, potato and chihuahua flowers were significantly increased as a function of the exposure time and increasing FA concentrations (Iqbal et al. 2021a; Wang et al. 2020; Fung et al. 2019; Dong et al. 2014; Sapko et al. 2011). In addition, higher MDA production was observed at the early stage of fungal infection after 2 h, which then gradually decreased until 6 h and finally started to increase again until day 7 after treatment with FA-producing fungi (Antić et al. 2020). Similarly, the toxic effects of FA also increased TBARS production, especially in tomato leaves, depending on the exposure time and concentration of FA (Singh and Upadhyay 2014, 2016). These observations suggest that FA toxicity can induce lipid peroxidation, which disrupts important cellular functions and reduces cellular stability. In addition, the disruption of biological functions of membranes, such as cell signalling, was found to increase the damage to cellular membranes in FA-treated plants as compared to control plants.

## FA-induced effects on hormone signalling

Pathogen attack can trigger defence or resistance mechanisms in various plants. The major phytohormones, such as salicylic acid (SA), jasmonic acid (JA) and ethylene (ET), induce signal transduction via complex signalling pathways (Zhang et al. 2022; Ederli et al. 2021; Dhar et al. 2020). The defence responses mediated by SA- and JA-dependent signalling mechanisms are triggered against biotrophic and necrotrophic pathogens, respectively. However, the defence mechanisms against hemi-biotrophic and necrotrophic fungal species are much more complex (Palazzini et al. 2018). It is interesting to note that SA and JA compete with each other in the resistance responses to infections (Poór et al. 2017; Denancé et al. 2013). Higher ROS levels induced by phytotoxins can lead to increased SA production, resulting in HR-like cell death at the site of infection to prevent the spread of infection (Ding and Ding 2020; Poór et al. 2017). Already low levels of SA can initiate a systemic acquired response for additional defence mechanisms against pathogenic attack, leading to the induction of numerous antimicrobial pathogenesis-related proteins (Iqbal et al. 2021b). Besides SA, JA is also produced during pathogenic attacks or injury which plays an essential role in the synthesis of the antimicrobial plant defensin (PDF) and protease inhibitors, but it is also responsible for the positive regulation of plant cell death by altering oxidative metabolism (Coll et al. 2011). Interestingly, SA negatively affects ET signalling, and its biosynthesis under stress conditions (Poór et al. 2013).

The role of ET in FA-treated leaves of tomato plants was determined in a 72-h-long experiment (Singh and Upadhyay 2014). The results showed a significant increase in ET production upon FA exposure as compared to control plants. However, ET emission was the highest 6 h after the treatment, indicating the involvement of ET in the initiation of cell death under phytotoxin stress. Higher level of ET production was also related to enhanced ROS generation. Therefore, ET and ROS induce each other’s production, which eventually leads to cell death (Singh and Upadhyay 2014). Recently, Iqbal et al. (2021a) explained the crucial role of ET in defence progression in tomato leaves of two genotypes, Ailsa Craig (WT) and ET receptor *Never ripe* (*Nr*) mutant plants under FA exposure between of 0.1 and 1.0 mM after 24- and 72-h-long treatments. The results showed higher ET production after 72 h following FA treatment at higher concentrations, suggesting a time- and concentration-dependent effect of FA, and FA-induced ET production in tomato leaves. On the basis of these data, *Nr* leaves were more sensitive to FA phytotoxicity, as compared to WT plants (Iqbal et al. 2021a). In parallel, another study reported the effect of FA (200 mg/L) on the expression of six genes encoding ET responsiveness in *Benincasa hispida* seedlings and found higher induction of four transcription factors, ACC oxidase and ET insensitive-like 1 protein isoform X1, suggesting the involvement of ET in modulating and regulating FA-induced oxidative stress (Wang et al. 2021). Nevertheless, further research is required for a comprehensive understanding of hormonal signalling under phytotoxin exposure.

## FA-elicited physiological effects

FA-induced phytotoxicity can affect the osmotic pressure, ion flux, micronutrient uptake, water potential, and it can result in the reduction of stomatal size, alterations in membrane permeability and cell death processes in plants (Wang et al. 2015; Dong et al. 2012; Bouizgarne et al. 2004). These stomatal abnormalities affect respiration and photosynthesis as well (Iqbal et al. 2021a). It has been reported that FA exposure mainly affects the electron transport chain, enzymatic activities and CO_2_ fixation (Singh et al. 2017). However, Wang and co-workers (2013, 2015, 2020) conducted experiments on cucumber leaves, and revealed also the toxic effects of FA on the photosynthetic process in a time- and FA concentration-dependent manner. Furthermore, FA stress leads to mitochondrial dysfunction due to higher levels of oxidative stress, leading to the initiation of cell death (Liu et al. 2020). For example, *Dendrobium sonia* 28 strain showed a higher mortality rate and growth reduction upon FA exposure (Dehgahi et al. 2016). Similarly, a high dose of FA exposure inhibited root growth and development of maize seedlings (Spss and Oliveira 2009). Likewise, the cell suspension cultures of *Nicotiana tabacum* treated with 100 µM of FA phytotoxin showed dilation of the endoplasmic reticulum and damage to cisternae (Jiao et al. 2013). In addition, FA-induced toxicity (1 mM) reduced root cell viability and adversely affected plasmodesmata and mitochondrial structure (Pavlovkin et al. 2004).

However, a number of other plant species have also been shown to be damaged by FA and the disease symptoms were associated with *Fusarium* infection. For example, tobacco cell suspensions showed significantly higher H_2_O_2_ production, lipid peroxidation and mitochondrial dysfunction under 100 µM of FA exposure after 24 h (Jiao et al. 2014). Similarly, FA treatment of *Arabidopsis* seedlings reduced root and root hair growth along with induction of membrane hyperpolarisation (Bouizgarne et al. 2006b). Similarly, FA-induced physicochemical changes such as reduced photosynthetic activity and plant biomass, turgor loss, as well as high proline content were observed in cape gooseberry plants at 100 mg/L FA concentration (Mendoza-Vargas et al. 2021). In addition, higher oxidative stress and antioxidant activity and reduced cell viability were observed in tomato cell suspensions at 2 × 10^−4^ M dose of FA. These FA-induced effects were enhanced with prolonged duration of FA exposure from 1 to 48 h (Kuźniak 2001). Furthermore, Hashem et al. (2021) documented the toxic effects of FA-producing *Fusarium* sp. on tomato plants showing wilting symptoms, reduction in chlorophyll content and plant growth. In addition, FA treatment (0.1–0.5 mM) in potato shoots significantly reduced the plant growth and length in a 4-week-long experiment (Arici and Meryem 2017). Surprisingly, in chihuahua plants (*Tacitus bellus* L.) infected with FA-producing *Fusarium* species, MDA levels were significantly reduced in a time-dependent manner together with high oxidative stress (Antić et al. 2020). In addition, wheat and faba bean plants showed growth inhibition under FA exposure when grown separately, but intercropping significantly reduced FA toxicity (Li et al. 2021). FA exposure at various doses can result in a variety of physiological and biochemical changes in different plant species, as well as impaired plant growth and development. However, these FA-mediated toxic effects depend on the plant species, the FA concentrations and the targeted plant organs in addition to the duration of exposure. In addition, plants resistant to FA-producing *Fusarium* species showed less toxicity symptoms and other physiological effects as compared to *Fusarium*-susceptible plants (Bouizgarne et al. 2004; Barna et al. 2011; Xie et al. 2011). These findings can also be applied to other phytotoxins to study their effects on plant growth and development, as well as metabolic processes (Table 1).

**Table 1:** Time- and concentration-dependent physiological effects of FA in different plant species

Plant species	Targeted plant organ	FA concentrations	Duration	Basic effects	References
*Arabidopsis thaliana* L.	Cell suspension	10^–8^–10^–3^ M	24 h	Enhanced ROS production, higher Ca^2+^ concentration, induction of phytoalexin synthesis, increased anion currents	Bouizgarne et al. (2006a)
*Benincasa hispida* L. (wax gourd)	Roots	75 μM	8 h	Changes in extensin levels were detected	Xie et al. (2011)
*Benincasa hispida* L. (wax gourd)	Seedlings	200 mg/L	2 days	Oxidative burst and induction of ET biosynthesis	Wang et al. (2021)
*Citrullus lanatus* L. (watermelon)	Leaves	0–100 mg/L	12 h	Inhibited N uptake, reduced leaf amide and protein content	Wu et al. (2008a, b)
*Citrullus lanatus* L. (watermelon)	Leaves	0–400 mg/L	48 h	Wilted cotyledons, necrosis, chlorosis and reduced number of lateral roots	Wu et al. (2008a)
*Citrullus lanatus* L. (watermelon)	Leaves and roots	0–400 mg/L	24 h	Wrinkled and wilted leaves, inhibited dehydrogenase activity of roots and reduced cell membrane potential	Wu et al. (2008b)
*Crocus sativus* L. (saffron)	Root tip cells	25–200 µM	60 h	Cell death induction, chromatin condensation, DNA fragmentation and H_2_O_2_ accumulation	Samadi and Shahsavan Behboodi (2006)
*Cucumis sativus* L. (cucumber)	Leaves	0–200 ppm	9 h	*Fusarium* wilt symptoms, stomatal closure, enhanced plant temperature and water loss	Wang et al. (2013)
*Cucumis sativus* L. (cucumber)	Leaves	0–100 ppm	9 h	Wilting, membrane injury and water loss	Wang et al. (2014)
*Cucumis sativus* L. (cucumber)	Plants Leaves and roots	Conidial suspension 10^6^ conidia/mL for 2 h and 0–100 ppm	8 days 9 h and 10 h	Reduced water absorption and stem hydraulic conductance, increased water loss	Wang et al. (2015)
*Dendrobium sonia*	Protocorm-like bodies (PLBs)	0.05–0.2 mM	4 weeks	High mortality of PLBs and growth reduction	Dehgahi et al. (2016)
*Gladiolus grandiflorus* L. (sword lily)	Gladiolus corms	Spore concentration 8 × 10^6^/mL	15 days	Lesion formation, increased FA production in tissues	Nosir et al. (2011)
*Hordeum vulgare* L. (barley)	Plants	5 × 10^−3^ M	20 h	Lesions formation, chlorosis, necrosis and wilting	Barna et al. (2011)
*Lilium *sp. (Lily cultivars Connecticut King and Star Gazer)	Lily bulblets	0.01, 0.1 and 1 mM	30 days	Fol-susceptible Stargazer lily accumulated more FA in tissues than Fol-resistant Asiatic hybrid lily, lily basal rot disease	Curir et al. (2000)
*Musa paradisiaca* L. (Gross Miche, *Musa* spp. AAA group)	Seedlings	4 × 10^6^ spores/mL for 2 h and then 5 mL conidia suspension in a barrel	15 days	Chlorosis, decreased transpiration rate and reduced stomatal conductance	Dong et al. (2012)
*Musa paradisiaca* L. (Gross Miche, *Musa* spp. AAA group)	Seedlings	4 × 10^6^ spores/mL for 2 h and then 5 mL conidia suspension with 400 g of quartz	15 days	Leaf senescence, reduced photosynthesis and stomatal conductance	Dong et al. (2014)
*Musa paradisiaca* L. (Gross Miche, *Musa* spp. AAA group)	Banana plantlets	0.5–1 mM	1 day	Discoloration of rhizome, pseudostems, wilting of leaves and rot of pseudostems	Ding et al. (2018)
*Nicotiana tabacum* L. (tobacco)	Cell suspensions	100 µM	24 h	Cell death, dilated endoplasmic reticulum, cisternae, chromatin condensation, DNA fragmentation	Jiao et al. (2013)
*Nicotiana tabacum* L.	Cell suspensions	100 µM	24 h	H_2_O_2_ overproduction, mitochondrial dysfunction and lipid peroxidation	Jiao et al. (2014)
*Orobanche ramosa* L. (hemp broomrape) and *Arabidopsis thaliana* L.	Seedlings	10^–7^–10^–3^ M	3 weeks	Reduced root and root hair growth, membrane hyperpolarization	Bouizgarne et al. (2006b)
*Phoenix dactylifera* L. (date palm)	Root hairs of seedlings	10^–7^–10^–3^ M	30 days	Altered membrane permeability and potential	Bouizgarne et al. (2004)
*Physalis peruviana* L. (cape gooseberry)	Plants	100 mg/L	12 h	Decreased photosynthetic rate, proline accumulation, turgor loss, reduced biomass	Mendoza-Vargas et al. (2021)
*Ricinus communis* L. (castor bean)	Root cells	1 mM	21 days	Decreased viability of root cells, affecting plasmodesmata and mitochondria	Pavlovkin et al. (2004)
*Solanum lycopersicum* L. (tomato)	Cell suspensions	2 × 10^–4^ M	1–48 h	Reduced cell viability, enhanced oxidative burst and higher antioxidant activities	Kuźniak (2001)
*Solanum lycopersicum* L.	Leaves	50–300 µg/mL	72 h	Formation of necrotic lesions, lipid peroxidation and DNA degradation	Singh and Upadhyay (2014)
*Solanum lycopersicum* L.	Leaves	50–300 µg/mL	72 h	Leaf wilting, cell death, high ROS levels, cellular damage and high SA content	Singh and Upadhyay (2016)
*Solanum lycopersicum* L.	Leaves	50–300 µg/mL	72 h	*Fusarium* wilt, cell death, high electrolytic leakage, necrosis, reduced chlorophyll content	Singh et al. (2017)
*Solanum lycopersicum* L.	Plants	10^6^ CFU (colony forming unit)	6 weeks	Reduced plant growth, wilting, reduction in chlorophyll content	Hashem et al. (2021)
*Solanum lycopersicum* L. (Ailsa Craig and *Never ripe* mutant)	Leaves	0.1 and 1 mM	24 and 72 h	Reduced chlorophyll content, reduced stomatal conductance and photosynthetic rate, cell death	Iqbal et al. (2021a)
*Solanum tuberosum* L.	Cell suspensions	10^–8^–10^–3^ M	10 min to 48 h	Decreased cell viability, induced toxicity to cells and enhanced oxidative burst	Sapko et al. (2011)
*Solanum tuberosum* L. (potato)	Shoots	0.1–0.5 mM	4 weeks	Decreased plant growth and length, reduced plantlets wet weight	Arici and Meryem (2017)
*Tacitus bellus* L. (chihuahua flower)	Plants	100 μL inoculum of 10^2^ spore suspension	24 h and 7 days	High oxidative burst, decreased MDA level	Antić et al. (2020)
*Triticum aestivum* L. (wheat) *Vicia faba* L. (faba bean)	Seedlings	50–200 mg/L	25 days	Inhibited growth of faba beans, intercropping increased physiological resistance and reduced FA phytotoxicity	Li et al. (2021)
*Zea mays* L. (Maize)	Seedlings	0.1–5 mM	8 days	Inhibited development of corn seedlings and reduction of root length	Spss and Oliveira (2009)

## FA-induced DNA fragmentation and genotoxicity

To maintain cellular homeostasis, plant cell death under toxin exposure or fungal infection eliminates old, damaged or diseased plant cells in a highly coordinated manner. In addition, cell death plays an essential role in the coordination of plant growth and development, as well as in the induction of cellular responses to various stresses (Valandro et al. 2020). Plants induce cell death at the infection site to prevent further spread of pathogen infection and toxin production (Iqbal et al. 2022; Xing et al. 2013). Saffron root tip cells were exposed to 25–200 µM FA for 60 h, and it was observed that FA induced higher H_2_O_2_ accumulation, chromatin condensation and DNA breakage followed by cell death (Samadi and Shahsavan Behboodi 2006). The maximum DNA fragmentation (50–60%) was detected at 25–100 µM FA over the different time periods, but less DNA fragmentation was observed at 200 µM of FA exposure. Furthermore, DNA fragmentation was suppressed by the activation of various inhibitors such as serine protease and caspase (Samadi and Shahsavan Behboodi 2006). FA exposure also resulted in the condensation of chromatin material (spheres budding from the nucleus) and the release of cytochrome* c* from mitochondria into cytosol (Samadi and Shahsavan Behboodi 2006). FA toxicity was also reported in tobacco cell suspension exposed to 100 µM FA for 24 h, showing high production of NO leading to cell death, dilated endoplasmic reticulum, chromatin condensation and DNA fragmentation (Dehgahi et al. 2016). Similarly, FA exposure (50–300 µg/mL) caused lipid peroxidation and formation of necrotic lesions in tomato leaves, followed by DNA degradation. FA infiltration into tomato leaves resulted in DNA fragmentation on agarose gel, whereas DNA fragmentation was absent in the case of control leaf tissues (Singh and Upadhyay 2014). Therefore, it can be concluded that FA toxicity may have detrimental effects on the control of defence-related genes engaged in hormonal signalling pathways (e.g. JA, ET and SA) regulated by ROS and NO, as well as cause DNA damage or fragmentation by activating the proteases in the cytosol upon FA exposure, which may contribute to cell death.

## Approaches to control FA toxicity

### Application of metal ions

FA toxicity can affect various physiological, biochemical and metabolic processes at the molecular level, which can hinder plant growth and development resulting in crop yield loss. Several strategies have been used to moderate or reduce FA toxicity in plants (Wang et al. 2020). For example, the use of exogenous zinc (Zn) and copper (Cu) increased the tolerance of cucumber plants to FA toxicity (Wang et al. 2020). The results showed that both Zn and Cu significantly reduced the deleterious effects of FA exposure, and altered the antioxidant activity. Application of these metals reduced wilting symptoms, cell membrane damage of roots and leaves, and promoted the plants’ growth and photosynthetic activity (Wang et al. 2020). In addition, Zn and Cu alleviated FA toxicity by 17% and 20%, respectively, by reducing the H_2_O_2_ production and lipid peroxidation (Wang et al. 2020). Furthermore, both metal treatments enhanced antioxidant enzymatic activities (SOD, CAT and POD) by preventing FA-induced oxidative burst (Wang et al. 2020). Cu and Zn have a significant influence on FA tolerance in cucumber plants by modifying the uptake and transport of FA in plants (Wang et al. 2020).

### Biological detoxification

Several bacterial and fungal species have been tested to detoxify or degrade FA. Interestingly, some antagonistic fluorescent strains of *Pseudomonas *sp. showed resistance to FA at a concentration of 500 ppm (Fakhouri et al. 2003). In addition, a non-pathogenic fungal strain of *Colletotrichum* sp. was found to be more efficient in detoxifying FA (200 ppm) after 4 days of treatment. This fungal strain degraded FA to 4-butyl-2-carboxypyrimidine, which had no toxic effects on tomato seedlings and fungal spore germination. However, *Colletotrichum *sp. was unable to degrade the high concentration of FA (400 ppm) and showed no growth even after 10 days of treatment (Fakhouri et al. 2003). Maina and co-workers (2008) used three bacterial strains, namely *Pseudomonas putida* 53, *Pseudomonas fluorescens* T58 and *Bacillus sphaericus* B43, to reduce the toxic effects of FA on tomato plants. These bacterial strains activated induced systemic resistance (ISR) to reduce the damage caused by *F. oxysporum* f. sp. *lycopersici*, which produces FA in tomato plants. The results showed that the H_2_O_2_ accumulation, chlorophyll degradation and ion leakage were significantly reduced in bacteria-treated plants as compared to control plants*.* In addition, SOD and GPX activities were reduced following bacterial treatment. However, ROS such as O_2_^**.**−^ showed higher levels in bacteria-treated plants in a time-dependent manner, eventually leading to membrane damage (Maina et al. 2008). These results clearly showed that ISR caused by biocontrol agents may be relevant to prevent FA-related damage in plants and serve as a basis for further studies to elucidate the unidentified mechanisms of action of biocontrol agents.

In addition, another fungal strain, *Mucor rouxii*, has shown the potential to degrade toxic FA to 8-hydroxyfusaric acid, a less toxic compound in *Gossypium hirsutum* and *G. barbadense* (Crutcher et al. 2017). The hydroxylation of the butyl chain of FA resulted in detoxification, and showed less phytotoxic effects in cotton plants as compared to the parent compound (FA) (Crutcher et al. 2017). Interestingly, controlling the genes responsible for this hydroxylation could be helpful in developing biocontrol agents to reduce FA toxicity (Crutcher et al. 2017). Similarly, the *Burkholderia ambifaria* T16 strain, which was isolated from the rhizosphere of barley, was shown to detoxify FA in barley seedlings and to rely solely on FA for its carbon, nitrogen and energy sources. Inoculation of barley seedlings with this bacterial species increased cell viability, cell density, indole-3-acetic acid production and biofilm formation (Simonetti et al. 2018). Furthermore, *B. ambifaria* T16 treatment also improved siderophore production, ACC deaminase activity and resistance to phytopathogenic *Fusarium *sp. (Simonetti et al. 2018). These results confirm the important role of *B. ambifaria* T16 in FA detoxification, and open up new avenues for the development of gene-modified FA detoxification technologies. A recent study also reported the conversion of FA into less toxic compounds, when the FA-producing fungus *F. verticillioides* was co-cultured with another fungus, *Gliocladium roseum* (Kuang et al. 2022). Using nuclear magnetic resonance (NMR) and mass spectrophotometry, Kuang et al. (2022) detected four different FA products in the co-culture inoculation and identified them as 4-(5-butylpicolinamido)butanoic acid (45BBA), 5-butylpyridine-2-carboxylic acid methyl ester (5B2CAM), bis(5-butyl-2-pyridinecarboxylate-N1,O2)-copper (B52P) and methyl 4-(5-butylpicolinamido)butanoate (M45BBA). These FA derivatives were tested on *Botrytis cinerea* and *Aspergillus niger* and showed reduced toxicity as compared to the parent compound FA. The 45BBA derivative of FA showed the lowest toxicity among tested FA derivatives (Kuang et al. 2022). Therefore, these results indicate the efficient antagonistic interaction between these fungal species leading to the biotransformation or detoxification of FA (Brauer et al. 2019).

## Future prospects

In addition to computational and experimental techniques, recent developments in “omics” approaches such as proteomics, transcriptomics, metagenomics, lipidomics and metabolomics have provided us new opportunities to search for novel bioactive fungicides for the agricultural sector in order to reduce crop losses and increase crop productivity (Brauer et al. 2019). For example, epigenetic approaches could use RNA interference such as bidirectional trafficking of plant–pathogen miRNAs to control pathogens and toxin production (Ratajczak and Ratajczak 2016). Similarly, metabolomics helps to study microbial interactions with plants and identify novel bioactive biomarkers with high efficacy and pathogen specificity to control pathogenic fungi (Johanningsmeier et al. 2016). Similarly, the integrated approach of metagenomics could play an essential role in the production of antifungal drugs, using an integrated method of metabolite evaluation and molecular library screening to select a drug candidate through the application of computational models (Brauer et al. 2019). However, more research is needed to find new antifungal molecules that can solve the problem of antifungal drug resistance in plants. The development of new chemicals against fungal species is a challenging task because of the time and resources required, as well as the need for accurate identification and characterisation of candidate compounds. The discovery of new antifungal molecules is far less frequent than the emergence of fungal species resistant to antifungal drugs. In addition, modification of the genome of economically important crops, either by breeding or transgenesis could be useful to control *Fusarium* diseases and their potential phytotoxins, including FA. The nature of plant tolerance or resistance to fungal infection, which can reduce crop yield and produce fungal resistant crops under biotic stress conditions, therefore needs to be understood in great detail. In addition, to advance the scientific understanding of FA phytotoxicity and its hidden host plant infection mechanism, the present study will be useful in understanding the long-term control of the phytotoxin. Further research on the synergistic effects of two or more phytotoxins is also needed to understand their phytotoxic effects in cross-kingdoms. To promote biocontrol of fungal diseases caused by FA, additional knowledge is needed to screen biocontrol strains that secrete siderophores and other metal chelating agents, such as iron supplementation to limit FA production and the growth of fungal pathogens.

## Conclusion

Exposure to FA increases oxidative stress via the enhanced accumulation of ROS, and affects both enzymatic and non-enzymatic antioxidant processes. FA-mediated ROS production also causes changes in hormonal signalling, photosynthetic apparatus, cellular structure and DNA damage. These FA-induced changes include mitochondrial dysfunction, lipid peroxidation, chlorosis and ultimately impaired plant growth and development, resulting in loss of crop production. However, various strategies such as the use of metals, such as Zn and Cu, co-culture inoculation with biocontrol agents and control of the FA biosynthesis genes in fungal species are being used to control FA production and reduce its effects in various crops.
